# Re-contacting participants for inclusion in the database of Genotypes and Phenotypes (dbGaP): Findings from three case-control studies of lung cancer

**DOI:** 10.1186/s13073-014-0054-x

**Published:** 2014-07-23

**Authors:** Michele L Cote, M Jay Harrison, Angela S Wenzlaff, Ann G Schwartz

**Affiliations:** Department of Oncology, Wayne State University School of Medicine, 4100 John R. Mailstop: MM04EP, Detroit, Michigan 48201 USA; Karmanos Cancer Institute, Population Studies and Disparities Research Program, Detroit, MI USA; Center for Molecular Medicine and Human Genetics, Wayne State University School of Medicine, Detroit, MI USA

## Abstract

**Background:**

Since January 2008, the National Institutes of Health (NIH) has required that all investigators who receive NIH support submit de-identified high-throughput genomic data to the database of Genotypes and Phenotypes (dbGaP). The purpose of this study was to explore the feasibility of re-consenting participants from three inactive studies, conducted from 2000 through 2009, to submit their data to dbGaP.

**Methods:**

Participants were those enrolled in one of three prior population-based case-control studies of lung cancer who had given a DNA sample. Consent to release de-identified data to dbGaP took place via mailed forms and follow-up phone calls. Chi-squared tests were used to examine differences in re-contact and consent proportions between groups.

**Results:**

A total of 2,471 participants were initially eligible for re-contact. Six hundred and thirty-eight participants were found to be deceased (n = 627) or did not give permission to re-contact (n = 11). Of the 1,833 remaining participants, 42.3% provided written consent, 37.0% could not be located, 13.7% verbally agreed to have their data released but never returned written consent, 5.3% refused, and 1.6% were too ill at the time of contact. There were significant differences in ability to locate participants by age, race, gender, and case-control status; however, once located, there were no differences in re-consent rates.

**Conclusion:**

This study demonstrates that while most previous study participants agreed to release data, a small proportion are opposed to submitting their data to dbGaP. In addition, it demonstrates the difficulty studies based on existing samples may have in locating inactive participants for re-consent.

## Background

Starting in January 2008, the National Institutes of Health (NIH) has required NIH-supported studies which generate high-throughput genomic data to submit their de-identified data to the database of Genotypes and Phenotypes (dbGaP) [1,2]. Many current genome-wide association studies (GWAS) include individuals who have only consented to aims of the original study, meaning that it may be necessary to re-contact participants to receive their consent for this additional use of their data. The only published study to assess the outcomes of re-consenting GWAS participants for this purpose is from the Adult Changes in Thought (ACT) study, a longitudinal cohort study of ageing and dementia [3]. In this study, of the 1,340 participants eligible for re-consent, 1,159 (86%) agreed to submit their data to the NIH central repository. A portion of the individuals who consented were also asked their views about the re-consenting process. The majority of respondents (69%) reported that it was very important that their permission was asked. Many respondents considered alternatives to consent, such as notification-only or opt-out, to be unacceptable (67% and 40%, respectively) [3].

Unlike the ACT study which maintained active relationships with participants during the time of their re-consent, this current study examined the success of re-consenting subjects who have had little or no contact with research investigators for up to 10 years. The original aims of the three case-control studies included in this analysis were to identify candidate genes or regions associated with lung cancer. Similar to the ACT study, all three studies drafted their original consent forms prior to the NIH mandate that genomic research with NIH support be sent to the dbGaP. Here, we explored re-consent feasibility and the willingness of study participants to submit their genomic data to dbGaP.

## Methods

Subjects were enrolled in one of three prior Wayne State University case-control studies: (1) the Family History study (FHS), which focused on individuals aged less than 50 years at lung cancer diagnosis; (2) the Women’s Epidemiology of Lung Diseases (WELD) study, which enrolled women with adenocarcinoma of the lung; and (3) the Exploring Health, Ancestry, and Lung Epidemiology (EXHALE) study, a study of African Americans with lung cancer [4]. These studies all received full board review and approval from the Wayne State University Institutional Review Board. All cases were identified through the Metropolitan Detroit Cancer Surveillance System (MDCSS), a part of the Surveillance, Epidemiology and End Results (SEER) program. Controls in the metropolitan Detroit area were identified through random digit dialing (FHS and WELD) or were volunteers (EXHALE). All controls were frequency matched to cases by 5-year age group, sex, and self-reported race. We attempted to re-consent participants who had given a blood sample in the original study to allow future GWAS data to be submitted to an NIH central repository, dbGaP. Eligible participants were those who were not known to be deceased at the start of the re-consent process and those who had indicated permission to be re-contacted on their original consent form. The process of consenting eligible participants started in February 2010. Data collected before 24 March 2011 were included in this analysis.

### Original study details

The FHS had DNA available for 141 cases and 250 controls from 15 September 1990 to 30 September 2003. Cases were defined as those aged less than 50 years (mean age, 42.1 years) with a primary neoplasm of the lung or bronchus. There were an equal number of men and women, and 73.4% (n = 287) of the study population self-identified as white and 22.0% (n = 86) as African American, with 4.6% (n = 18) identifying as other races.

The WELD study recruited 530 cases and 529 controls who provided a DNA sample. Cases were defined as women (age range, 18-74 years) diagnosed with primary non-small cell lung cancer (NSCLC) between November 2001 and October 2005. The racial make-up of the sample population was 76.7% (n = 812) white, 21.2% (n = 224) African American, and 2.2% (n = 23) other races.

The EXHALE study recruited 464 African American cases and 557 African American controls prior to revising the consent form to allow for dbGaP submission at the time of the initial consent. Cases diagnosed with lung cancer of any histology from 1 November 2005 to 31 October 2009 were eligible for the study. The study population was 47.0% men and 53.0% women.

### Re-consenting process: mail

The names and social security numbers (when available) of all subjects were linked to the MDCSS to identify deceased individuals. Current addresses and telephone numbers of participants who were not thought to be deceased were obtained from MDCSS records, or through Lexus Nexis, which provides a search engine to identify past and current residential addresses and telephone numbers based on applications to credit agencies. Those eligible for re-contact (that is, not known to be deceased and allowing future contact) were mailed an introductory letter reminding the subject of their previous study participation and outlining the request for additional consent to release their data to dbGaP. The packet also contained two copies of the new consent form and a postage paid envelope, with the request to mail back the signed consent form indicating whether they would agree to release their data. The four-page consent form described the study procedures as follows: ‘Allow us to submit both genetic and risk factor information (age, gender, tobacco exposure, health status, etc.) to the NIH central data repository. These data will not include your name, address, phone number or any other usual identifiers.’

### Re-consenting process: telephone follow-up

If the consent form was not returned after 1 month, an interviewer followed up with a telephone call. The interviewer tried the telephone number at various times during morning, afternoon and evening hours during the week and on Saturdays for 2 to 3 months. If the provided phone numbers were incorrect or disconnected, alternate phone numbers were tried, if available. Consent letters identified by the participant as lost, thrown away, or never received were resent to a confirmed address. Four interviewers conducted the participant phone calls and were the same interviewers who had conducted most of the participant interviews during the original studies. An effort was made to match participants to their original study interviewer. Upon reaching a participant on the phone, the interviewers summarized the introductory letter, asked the participant if they had received the letter, explained the consent process, and asked about plans to mail the letter back. Interviewers logged the calls made to each participant. For the analyses of the number of phone calls, letters sent to each participant and detailed dialog with the participant, a randomized sample of 105 call logs, 35 from each study, were used.

The participant’s re-consent results included: the date the re-consent letter was sent, the date the re-consent form was returned, the decision to participate (yes or no), as well as the reasons a consent form was not returned (invalid address, invalid phone number, both, and so on). A limited amount of demographic information (age, race, and sex) collected during the original studies was also available for each subject. Chi-squared tests were used to examine differences in re-contact and consent proportions between groups, and a *P* value of <0.05 was considered statistically significant. These data were analyzed using SAS version 9.2.

## Results

### Mail contact

**Figure 1:**
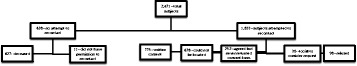
**Outcome of re-contacting 2,471 case and control participants from three population-based lung cancer studies.**

Figure 1 describes the outcome of the re-consenting process. Of the 2,471 participants in the original studies, no attempt was made to re-consent 638 individuals (n = 627 due to death and n = 11 because permission was not granted to be re-contacted). Of the 1,833 where re-contacted was attempted, 42.3% (n = 775) consented to have their data submitted to dbGaP, 37.0% (n = 678) could not be located, 13.7% (n = 252) told interviewers that they planned to return the consent form in the future but never did, 5.3% (n = 98) refused, and 1.6% (n = 30) were too sick to respond. Thus, a total of 1,402 (56.7%) of the original 2,471 study participants either consented (n = 775) or were deceased (n = 627) and could have their data submitted to dbGaP.

Our success at contacting living study subjects and obtaining consent to submit data to dbGaP is shown in Table 1. We attempted to contact 1,833 living former research participants. The availability of correct contact information varied significantly by study, gender, age, race, and case or control status. Women, those aged more than 50 years at the time of the original study, whites, and cases were significantly more likely to be contacted but these variables were not associated with consent to submit data to dbGaP (Table 1). Once contact was established, the study (*P* value = 0.76), gender (*P* value = 0.05), age (*P* value = 0.07), race (*P* value = 0.43), and case or control status (*P* value = 0.48) did not impact whether a participant was willing to consent to have their data submitted to dbGaP. Overall, 11.3% (98/871) of the former research participants who were located did not consent to release their data to dbGaP.

**Table 1:** **Living participants by re-contact status and consent**

	**Contacted** ^**a**^ **(n = 1,820)**			**Status of consent** ^**b**^ **(n = 871)**		
	**Yes**	**No**	**p-value**	**Given**	**Refused**	**p-value**
Study			<0.0001			0.76
FHS	138 (48.6%)	146 (51.4%)		98 (90.7%)	10 (9.3%)	
WELD	551 (73.3%)	201 (26.7%)		423 (88.7%)	54 (11.3%)	
INHALE	456 (58.2%)	328 (41.8%)		252 (88.1%)	34 (11.9%)	
Sex			<0.0001			0.05
Male	238 (48.5%)	253 (51.5%)		122 (84.1%)	23 (15.9%)	
Female	907 (68.3%)	422 (31.7%)		651 (89.7%)	75 (10.3%)	
Age (years)			<0.0001			0.07
<49	229 (51.0%)	220 (49.0%)		163 (92.6%)	13 (7.4%)	
50+	916 (66.8%)	455 (33.2%)		610 (87.8%)	85 (12.2%)	
Race			<0.0001			0.43
White	578 (72.2%)	223 (27.8%)		448 (89.8%)	51 (10.2%)	
Black	550 (55.6%)	439 (44.4%)		312 (87.2%)	46 (12.9%)	
Other	17 (56.7%)	13 (43.3%)		13 (92.9%)	1 (7.1%)	
Status			0.0009			0.48
Case	362 (68.8%)	164 (31.2%)		248 (89.9%)	28 (10.1%)	
Control	783 (60.5%)	511 (39.5%)		525 (88.2%)	70 (11.8%)	

^a^Excludes 13 people for whom contact status was unclear/still pending.

^b^Excludes 274 people who were successfully contacted and verbally agreed but never sent back the form (n = 252), individuals who were contacted but too sick to give informed consent (n = 30), and n = 2 individuals who were deceased but had next of kin return a consent form.

### Interviewer phone calls

Of the 105 phone call logs that were sampled, 370 phone calls were made by the interviewers. An average of 3.5 phone calls were made to each participant and there were 8.6 calls made per participant consent obtained. The number of calls per consent differed with 12.6 calls made per consent in the EXHALE study; 10.1 calls per consent in the FHSIII study; and 5.5 calls made per consent in the WELD study (data not shown).

## Discussion

A total of 56.7% of the original 2,471 study participants either consented or were deceased and therefore will have their data submitted to dbGaP. Our local IRB allows for submission of de-identified GWAS data from deceased individuals to be shared with dbGaP. Unfortunately, we could not locate over one-third of our former study participants. Of individuals contacted, 11.2% did not want their data released. The number refusing data submission in our study was similar to those in the Adult Changes in Thought (ACT) study. This study reported that of their 1,311 participants who were re-consented, 88.4% (n = 1,159) agreed to submit their data to the NIH central repository and 11.6% (n = 152) declined [3]. Unlike our study where all re-consenting took place through the mail or over the telephone, some (n = 353) of the re-consents in the ACT study were obtained during bi-annual face-to-face encounters between investigators and participants. Of these 353 participants, 9.6% refused participation while being consented in person [3]. This supports findings from our study that suggest a consistent minority of participants are opposed to submitting their data, regardless of study methodologies or disease site.

While no other studies are known to have examined the re-consent process, other studies have assessed the attitudes of the public toward participation in genetic cohorts and DNA data sharing. Focus groups with participants from a genetic study of epilepsy noted that participants felt genomic information should not be publically released without explicit consent from research participants [5]. A survey of the general U.S. adult population on their willingness to participate in a large (hypothetical) genetic cohort study in which participant DNA would be biobanked and made available to a wider scientific community reported widespread support (84%) but somewhat less agreement to participate (60%) [6]. Some prior studies have suggested that African Americans, women, older ages, lower income individuals, and individuals with less education are less likely to participate in genetic research or to allow their samples to be banked for future study [7–9], although these findings are not consistent [8]. Among individuals who responded to our re-consent request, our study did not find differences by age, race, gender, or case-control status.

The detailed call logs kept by each interviewer gave us the opportunity to qualitatively examine reasons given for not returning the re-consent form. Common themes included ‘forgetting to send back the form’ or misplacing/accidentally throwing the form away. One participant conveyed fears of their data being ‘hacked into through computer networks’ and others admitted that they generally did not feel comfortable submitting their data. These concerns are similar to those which have been previously identified [6]. In addition, nearly half of the call logs which contained dialog indicated that the participant had forgotten that they had participated in the original study. This is not uncommon, with a recent study reporting that over one-quarter of individuals who were recruited into genomic research studies did not recall signing informed consent documents, and more than half could not recall with whom they agreed to share their data [10].

Approaching research participants for additional permissions can be time-consuming and expensive [11]. We estimate that the overall cost of our re-consent process would be equal to the annual salary of one full-time interviewer, which (including fringe benefits and indirect costs) is roughly $47.60 per individual we attempted to re-contact. This is comparable to the Ludman et al. study which quoted $50 dollars per person, and employed a similar protocol of following the initial letter with a telephone call in the case of non-response [3]. In weighing the cost *versus* the benefit of the re-consent process the resources needed to replicate a study of this size must be considered. It is likely the ability to re-use these data is a much more economical approach when compared to obtaining new study participants.

At this point there is no universal consensus as to how participants in genomic research should be made aware of the potential future uses or sharing of the data generated from their biospecimens. One suggestion is to use a broad consent that allows continual research on collected samples. Essentially, broad consent describes the future deposition of genomic data into databases to allow secondary studies by other researchers, and may also include the distribution of biospecimens for additional studies [2]. While broad consent may bypass the difficulty in re-contacting participants to consent for secondary research, debate remains about participant autonomy with this approach [2,12]. In one study, the acceptability of broad consent was almost evenly split; with 42% of individuals in a biobank preferred to be asked permission for each research project separately rather than sign one consent form initially, while 48% would sign a broad consent form for future research [6]. Others suggest that broad consent and informed consent are not mutually exclusive, and may indeed be the most preferable method for genomic research [13]. Of course, broad consents constructed today may not include the necessary language specific to future projects and informed consent requirements, suggesting research in this area will be ongoing. Continuous engagement of research participants and the general public regarding the risks and benefits of genomic research has been recommended [14].

There are limitations to this analysis that should be considered. We could not locate over one-third of the participants from our lung cancer case-control studies. Due to the dates of enrollment into the original studies, some of the participants had not been contacted in 10 years. There is little doubt that increased time between an initial study and the re-consent process can present challenges in re-contacting participants. Age at original interview, sex, and race were all associated with the likelihood of obtaining a participant’s current contact information. Younger participants may be more transient due to changes in marital status and occupation. We also had more difficulty locating African Americans and men. It is difficult to interpret why these two demographics were more difficult to locate. In addition, 13.7% of participants who were re-contacted expressed that they planned to send in their re-consent form when questioned on the phone, yet had not done so after several months. While further telephone calls or reminders might be helpful, it is also possible these individuals represent ‘soft’ refusals, and do not want their information shared. Lastly, we were unable to draw conclusions about certain participant characteristics due to wide variations in eligibility criteria between the three studies. For example, it was difficult to determine the effects that time spent as an inactive participant had on willingness to consent, *versus* race, age, or gender.

## Conclusions

This study found a proportion of former study participants are opposed to submitting their genetic and health data to the NIH supported dbGaP database, which suggests these re-consent efforts may be worthwhile and ethically necessary when using data and specimens from past studies. It also demonstrates the difficulties future studies may have in locating inactive participants for re-consent, particularly in mobile and minority populations.
